# Abundant Taxa and Favorable Pathways in the Microbiome of Soda-Saline Lakes in Inner Mongolia

**DOI:** 10.3389/fmicb.2020.01740

**Published:** 2020-07-24

**Authors:** Dahe Zhao, Shengjie Zhang, Qiong Xue, Junyu Chen, Jian Zhou, Feiyue Cheng, Ming Li, Yaxin Zhu, Haiying Yu, Songnian Hu, Yanning Zheng, Shuangjiang Liu, Hua Xiang

**Affiliations:** ^1^State Key Laboratory of Microbial Resources, Institute of Microbiology, Chinese Academy of Sciences, Beijing, China; ^2^College of Life Sciences, University of Chinese Academy of Sciences, Beijing, China

**Keywords:** soda-saline lakes, deep metagenomic sequencing, microbiome, abundant taxa, sulfur cycling, glucan metabolism

## Abstract

Soda-saline lakes are a special type of alkaline lake in which the chloride concentration is greater than the carbonate/bicarbonate concentration. Due to the high pH and a usually higher osmotic pressure than that of a normal soda lake, the microbes may need more energy to thrive in such a double-extreme environment. In this study, we systematically investigated the microbiome of the brine and sediment samples of nine artificially separated ponds (salinities from 5.5% to saturation) within two soda-saline lakes in Inner Mongolia of China, assisted by deep metagenomic sequencing. The main inorganic ions shaped the microbial community in both the brines and sediments, and the chloride concentration exhibited the most significant effect. A total of 385 metagenome-assembled genomes (MAGs) were generated, in which 38 MAGs were revealed as the abundant species in at least one of the eighteen different samples. Interestingly, these abundant species also represented the most branches of the microbiome of the soda-saline lakes at the phylum level. These abundant taxa were close relatives of microorganisms from classic soda lakes and neutral saline environments, but forming a combination of both habitats. Notably, approximately half of the abundant MAGs had the potential to drive dissimilatory sulfur cycling. These MAGs included four autotrophic *Ectothiorhodospiraceae* MAGs, one *Cyanobacteria* MAG and nine heterotrophic MAGs with the potential to oxidize sulfur, as well as four abundant MAGs containing genes for elemental sulfur respiration. The possible reason is that reductive sulfur compounds could provide additional energy for the related species, and reductions of oxidative sulfur compounds are more prone to occur under alkaline conditions which support the sulfur cycling. In addition, a unique 1,4-alpha-glucan phosphorylation pathway, but not a normal hydrolysis one, was found in the abundant *Candidatus* Nanohaloarchaeota MAG NHA-1, which would produce more energy in polysaccharide degradation. In summary, this work has revealed the abundant taxa and favorable pathways in the soda-saline lakes, indicating that efficient energy regeneration pathway may increase the capacity for environmental adaptation in such saline-alkaline environments. These findings may help to elucidate the relationship between microbial metabolism and adaptation to extreme environments.

## Introduction

A soda lake is a type of saline lake with extremely high pH and salinity mainly due to high concentrations (exceeding an equivalent percentage of 25) of carbonate/bicarbonate (Grant and Sorokin, 2011; Boros and Kolpakova, 2018). Recently, soda lakes were further divided into “soda” and “soda-saline” types based on the level of bicarbonate and carbonate. It is defined as “soda” type when the sum of bicarbonate and carbonate concentrations are the first in the rank of dominant ions, and is “soda-saline” type when the concentration of other ions is higher than that of bicarbonate/carbonate (Boros and Kolpakova, 2018). In these saline and alkaline environments, microorganisms exhibit surprisingly high biodiversity (Grant, 2006; Mesbah et al., 2007; Asao et al., 2011; Lanzen et al., 2013), relatively high primary productivity rates (Melack and Kilham, 1974; Melack, 1981; Kompantseva et al., 2009; Antony et al., 2013; Zorz et al., 2019), vigorous oxidation and reduction reactions of sulfur (Sorokin et al., 2010, 2011; Stam et al., 2010; Tourova et al., 2013; Vavourakis et al., 2019), and elevated metabolic activity of cellulose, methane, nitrogen and arsenic (Iversen et al., 1987; Carini and Joye, 2008; Oremland et al., 2017; Phitsuwan et al., 2019). High concentrations of inorganic ions, such as (bi)carbonate and phosphate provide adequate essential elements, while hydrogen sulfide exhibits low toxicity under alkaline conditions (Sorokin et al., 2015). This would support the microbes inhabiting such alkaline and saline environments, and playing important roles in the elemental cycling (Sorokin et al., 2014).

In the brines of alkaline soda lakes, *Bacteroidetes*, *Alphaproteobacteria*, *Gammaproteobacteria*, and *Euryarchaeota* were identified as taxa with the highest levels of abundance at different salinities (from 170 to 400 g/L) by both amplicon sequencing of the 16S rRNA gene and direct metagenomic sequencing (Vavourakis et al., 2016). The genomes of haloalkaliphilic members of the Candidate Phyla Radiation (CPR) and several hundred other novel prokaryote lineages were obtained from the metagenomic assembly of sequences from the sediment of soda lakes, and the Wood-Ljungdahl (WL) pathway for carbon fixation was detected in more taxa than already known groups from the same samples (Vavourakis et al., 2018). The autotrophic microbial community based on the detection of molecular markers, ribulose-1,5-bisphosphate carboxylase (RuBisCO) and ATP citrate lyase (Acl) in the Calvin-Benson-Bassham (CBB) and reductive tricarboxylic acid cycles, respectively, indicated that haloalkaliphilic cyanobacteria and sulfur-oxidizing bacteria of the genus *Halorhodospira* were predominant in soda lakes (Kovaleva et al., 2011; Tourova et al., 2011). Interestingly, even alkaline soda lakes separated by a large distance between Asia and North America share a similar core microbiome (Zorz et al., 2019).

As is well known, haloalkaliphiles from three domains of life thrive in the extreme environments with high salinity and alkalinity (Banciu and Muntyan, 2015). Monovalent cation/proton antiporters are widely present in archaea and bacteria and function in intracellular pH homeostasis (Krulwich et al., 2011). The biosynthesis or uptake of compatible solutes (such as glycine betaine and ectoine) by halophilic and haloalkaliphilic bacteria are commonly used as the primary mechanism to resist extracellular osmotic pressure (Roberts, 2005). Haloarchaea and anaerobic *Natranaerobiaceae* primarily maintain osmotic balance using K^+^ and Cl^–^ import systems (Gunde-Cimerman et al., 2018). Because of relatively low solubility and incomplete ionization of carbonate/bicarbonate (comparing with chloride), haloalkaliphilic microbes (prefer NaCl) seemed to resist more osmotic pressure than natronophilic ones (prefer NaHCO_3_/Na_2_CO_3_) under alkaline conditions (Sorokin et al., 2015). The microbes inhabiting soda lakes, especially the soda-saline type, are considered to be the ideal materials to research the environmental adaptation to high salinity and alkalinity. Importantly, microbes must consume a large amount of energy to maintain neutral cytoplasm and osmotic balance in response to extreme conditions (Banciu and Muntyan, 2015). The favorable metabolic pathways of abundant taxa to produce sufficient energy need to be followed through at the metagenomics level.

Hundreds of small soda lakes and pans are located in the Inner Mongolia Autonomous Region of China (Zheng et al., 2002). Analysis of the physicochemical factors in these lakes indicated that many of them are soda-saline lakes of the chloride-carbonate-sulfate type, providing an applicable system for studying the coupling of carbon and sulfur cycling and microbial environmental adaptation. In this study, we collected brine and sediment samples from nine ponds associated with two soda-saline lakes and performed deep metagenomic sequencing. Combined with environmental characterizations, we dissected the microbial community structures and relationships based on metagenomic reads and assembled genomes, subsequently focusing on the abundant species representing most branches of a phylogenomic tree. The efficient energy regeneration pathways in the abundant MAGs may increase the capacity for environmental adaptation in such saline-alkaline environments. The superiority of energy production and thermodynamics in the abundant species was assessed to further understand the metabolic mechanism of adaptation to such extremely alkaline and saline conditions.

## Materials and Methods

### Physicochemical Characterization

Salinity was measured using a handheld refractometer (Beijing Wanchengbeizeng Precision Instrument Co., Ltd., Beijing, China). Conductivity, pH and the concentrations of CO_3_^2–^ and HCO_3_^–^ were measured using a SensoDirect 150 and an MD600 Photometer (Lovibond^®^ Water Testing, Dortmund, Germany). The concentrations of other inorganic ions (Cl^–^, SO_4_^2–^, PO_4_^3–^, Mg^2+^, Ca^2+^, and NH_4_^+^) and total organic nitrogen were measured using an Aquakem^TM^ 250 discrete photometric Autoanalyzer (Thermo Fisher Scientific, MA, United States).

### DNA Extraction and Metagenomic Sequencing

Brine samples were prefiltered through four layers of gauze to eliminate eukaryotic animals and plants. The microorganisms in each sample were collected by 0.8- and 0.22-μm filters. Then, filters were used to extract DNA with a PowerWater^®^ DNA Isolation kit (MoBio, CA, United States). DNA extracted from the same sample was mixed as a single sample. Total DNA of sediment samples was extracted using a PowerSoil^®^ DNA Isolation kit (MoBio, CA, United States). The DNA concentration and purity were measured using a Qubit^®^ dsDNA Assay kit with a Qubit^®^ 2.0 Fluorometer (Life Technologies, CA, United States) and a NanoPhotometer^®^ spectrophotometer (IMPLEN, CA, United States), respectively. The OD_260_/OD_280_ values of the samples were 1.8∼2.0, and DNA concentrations were >1 μg. Library construction and shotgun sequencing were performed using an Illumina HiSeq-2000 platform (Illumina, United States) to generate 150-bp paired-end reads.

### Metagenomic Data Analysis Based on a Non-redundant Gene Catalog

Quality control of the raw reads was conducted using Readfq (V8^1^) to remove low-quality reads and ensure that (a) each read had no more than 40 bases with a quality score smaller than 38, (b) each read had less than 10 ambiguous nucleotides, and (c) no reads shared more than a 15-bp overlap with the adapter. Clean reads were assembled into contigs using MEGAHIT (v1.1.2) (Li et al., 2015) with the following parameters: –presets meta-large (-min-count 2, –k-min 27, –k-max 127, and –k-step 10). Unassembled read pairs were retrieved by mapping paired-reads to contigs using BBMap (v37.57)^2^ with the following parameters: kfilter = 22, subfilter = 15, and maxindel = 80. Coassembly was conducted using MEGAHIT with the same parameters described above to acquire low-abundance reads. Contigs from single-assembled samples and one coassembly were merged together, and contigs with <500 bp were removed (Qin et al., 2014).

Open reading frames (ORFs) were predicted using MetaGeneMark (GeneMark.hmm v3.38) (Zhu et al., 2010) with default parameters. ORFs with lengths <100 bp were removed to reduce the number of pseudogenes (Qin et al., 2014). Redundancy removal was executed using CD-HIT (v4.7) (Li and Godzik, 2006) with the following parameters: -c 0.95, -G 0, -aS 0.9, -g 1, and -d 0. Clean reads from the 18 samples were mapped to the nr-ORF catalog using BWA mem (v0.7.17) (Li and Durbin, 2010) with the default parameters. ORFs with <2 reads aligned from the 18 samples were removed to prevent incorrect assembly. The abundance of each non-redundant gene in one sample was calculated based on the proportion of the mapped number of reads (counted by BamM^3^) divided by the gene length (Qin et al., 2012). Taxonomy assignments were performed by mapping the amino acid sequences against the NCBI non-redundant database (Version: 20170923^4^) using Diamond (v0.9.10.111) (Buchfink et al., 2015) with the following parameters: –taxonmap, –taxonnodes, -e 1e-5, and –top 10. Non-redundant genes were assigned to the corresponding taxa calculated by the Lowest Common Ancestor (LCA) algorithm (Huson et al., 2007) in Diamond. The abundance of each metagenomic operational taxonomic unit (mOTU) (Sunagawa et al., 2013) was the sum of the abundances of all non-redundant genes assigned to that mOTU (Qin et al., 2012) and was supplied in Data Sheet 1 and 2. Functional annotation was performed using the KEGG databases by uploading to the Automatic Annotation Server (v2.1) (Moriya et al., 2007). The GENES dataset was set for Prokaryotes, while the Assignment method was set as BBH.

### Binning and Post Analysis of MAGs

Single sample assembly and coassembly of three samples with same type and similar salinity (water and sediment samples of HC5, DK15, and HC17; water and sediment samples of HC22, DK20, and HC26; water and sediment samples of DK32, DK27, and HC27; total 6 groups) were conducted to obtain low abundance contigs. Contig datasets were binned using MetaBAT (v2.12.1) (Kang et al., 2015) with default parameters (contigs of less than 2500 bp were discarded) based on the tetranucleotide frequency and coverage values obtained by mapping the clean data onto the contig datasets using BWA mem (v0.7.17) (Li and Durbin, 2010). Genome completeness and contamination were estimated using CheckM (v1.0.12) (Parks et al., 2015) to generate genomes satisfying the minimum information about a MAG (Bowers et al., 2017). Draft genomes were dereplicated according to ANI using dRep (v2.2.1) (Olm et al., 2017). Gene and protein-coding sequences were predicted using Prodigal (v2.6.3) (Hyatt et al., 2010). The abundance of each MAG in each sample was calculated, equaling the sum of the coverage of all contigs multiplied by their respective length and divided by the genome size. Taxonomic assignments for each bin were performed using CheckM, the Diamond aligned to UniProt TrEMBL database (Bateman et al., 2017), and PhyloPhlan (v0.99) (Segata et al., 2013) and were manually curated afterward. A phylogenomic tree was constructed using reference genomes based on the most conserved 400 proteins across bacteria and archaea using PhyloPhlan and visualized using iTOL (v4) (Letunic and Bork, 2019). Functional annotation was conducted using GhostKOALA (‘genus_prokaryotes + family_eukaryotes + viruses’; v2.0) (Kanehisa et al., 2016) to reconstruct the metabolic pathways and was supplied in Data Sheet 4. The direction of dissimilatory sulfur metabolism by DsrAB was determined by the present or absent of *dsrD* and *dsrEFH* (Supplementary Table 8) (Anantharaman et al., 2018), and these genes were predicted based on the HMM profile from TIGRFAM (Haft et al., 2003) and Pfam (El-Gebali et al., 2019) using Hmmscan v3.1b2 (Eddy, 2011). For the phylogenetic analysis, proteins assigned to *Ca.* Nanohaloarchaeota and *Ca.* Woesearchaeota were retrieved from the corresponding 19 MAGs and aligned using MEGA X (Kumar et al., 2018) with the reference protein alpha-glucan phosphorylase from *Escherichia coli* (PWL89129.1).

### Statistical Analysis

The datasets generated above were statistically analyzed using the free software R Project^5^. The sampling map was visualized using the leaflet package. The Shannon diversity indexes of samples were calculated using the vegan package^6^ and visualized by the ggpubr package^7^. Heatmap cluster analysis and principal component analysis (PCA) were visualized using the pheatmap^8^ and ggbiplot packages^9^, respectively. Redundancy analysis (RDA) was performed using the vegan package with all taxon abundances in genus level and physiochemical data. The co-occurrence network was based on the abundances of 385 MAGs across 18 samples (Supplementary Table 5). Pearson correlation coefficients were calculated using the psych package^10^, where a Pearson correlation coefficient > 0.9 and *p*-value < 0.01 were used. The network was visualized using Cytoscape (Shannon et al., 2003).

### Data Availability

The raw sequence reads of 18 metagenomes were deposited in NCBI (see foot note 4) with the projectID PRJNA549802 and in gcMeta (Shi et al., 2019) with the projectID NMDC10010899. The binning results can be accessed at figshare^11^.

## Results and Discussion

### Multiple Environmental Factors Shape Microbial Community Composition

Habor Lake (DK) and Hutong Qagan Lake (HC) are located in the southwest of Inner Mongolia Autonomous Region of China (Supplementary Image 1A) and comprise numerous artificial ponds with and a typical depth of 1–5 m. The ponds from same lake share the similar ratio of inorganic salts but different total salinities. Brine and sediment samples of four DK ponds (DK15, DK20, DK27, and DK32) and five HC ponds (HC5, HC27, HC17, HC22, and HC26) were collected (Supplementary Images 1B,C). The salinities of these ponds ranged from 5.5% to saturation with pH values greater than 9.8. CO_3_^2–^ and HCO_3_^–^ concentrations ranged from 78.33 to 820 mM and from 80.33 to 385.25 mM, respectively, while the chloride concentrations were 1.5–2.4 times as much as the sum of both. Therefore, both DK and HC were classified as soda-saline lakes of the chloride-carbonate-sulfate type (Boros and Kolpakova, 2018) and will simply be called soda-saline lakes in the text. The values of additional physicochemical parameters (magnesium ion, calcium ion, chloride, sulfate, phosphate, ammonia and total organic nitrogen concentrations) are shown in Supplementary Table 1.

Brine and sediment samples from the nine ponds were used to perform deep metagenomic sequencing (Supplementary Table 2). The average number of raw bases for the 18 samples was 15.98 Gb, and a total of 281.05 Gb of clean data was obtained after quality filtering. The bioinformatic analyses below were performed using these metagenomes.

**FIGURE 1:**
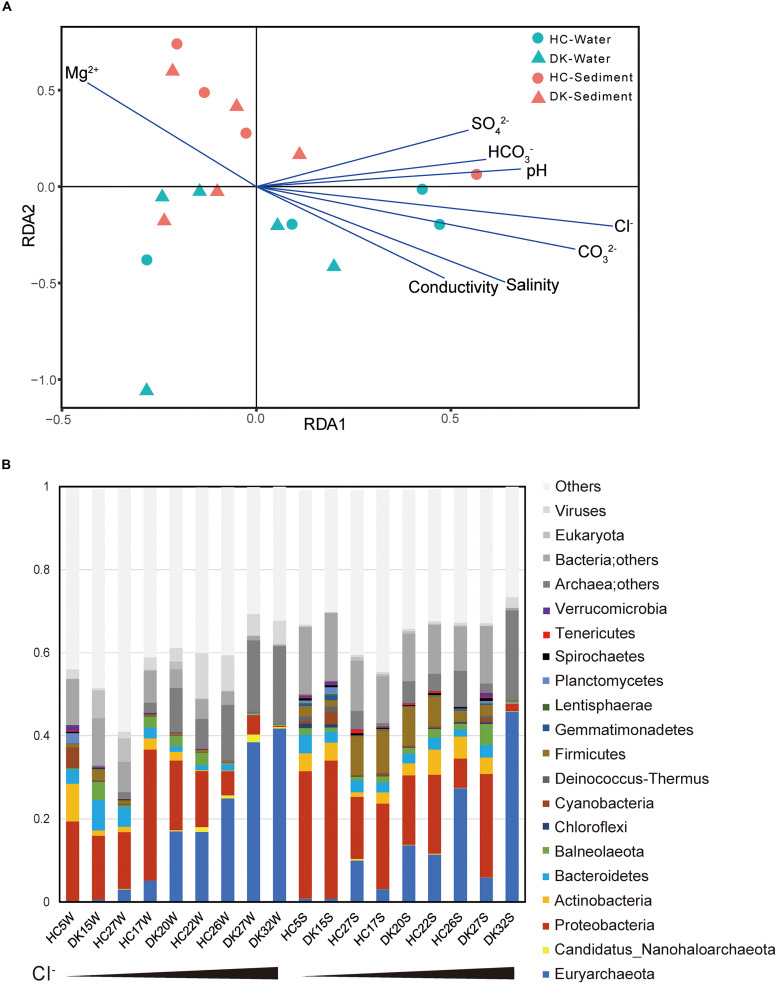
Relationship between environmental factors and microbial communities. **(A)** The influence of environmental factors on microbial community structure was analyzed by redundancy analysis. **(B)** The microbial composition of eighteen samples and the observed relative abundances (Supplementary Table 3) of taxa at the phylum level. The total relative abundances of taxa (greater than 0.1%) are shown. The first nine samples were water samples, whereas the last nine were sediment samples and were sorted by Cl^–^ concentration.

The microbial community composition was determined based on the taxonomy assignment of total non-redundant gene catalog acquired from metagenomics assembly, open reading frame (ORF) prediction and redundancy removal, and the influences of environmental factors were analyzed by redundancy analysis (RDA) based on the microbial composition of 18 samples at the genus level. The brine and sediment samples clustered together in the RDA (Figure 1A), suggesting that the microbial community structures in the two sample types were significantly different. This conclusion was also supported by the principal component analysis (PCA) results (Supplementary Image 3A), whereas the microbial community structures of similar types of samples from different lakes (DK and HC) were generally similar (Supplementary Image 3B). Regarding microbial diversity, the Shannon-Weaver index value obtained for the sediment was considerably increased compared with that observed for the brine (Supplementary Image 4A). The sediment ecosystems, especially the anoxic environments, may provide more opportunities for niche diversification (Vavourakis et al., 2018). Again, the overall biodiversity in the DK and HC was similar (Supplementary Image 4B). For physicochemical factors, the Cl^–^ concentration was the most influential environmental factor determining microbial composition (Figure 1A), to which pH, salinity, CO_3_^2–^, HCO_3_^–^, SO_4_^2–^, and conductivity were positively correlated. However, the Mg^2+^ concentration was negatively correlated (Figure 1A and Supplementary Image 2). The pH value, carbonate/bicarbonate concentrations and salinity appear to be of importance in shaping the prokaryotic communities in salt lakes (Pagaling et al., 2009; Simachew et al., 2016). As shown in Supplementary Image 2 and Figure 1, the Cl^–^ concentration, which is positively correlated with pH, salinity, CO_3_^2–^, HCO_3_^–^, SO_4_^2–^ and conductivity, was a key factor affecting microbial communities among these saline-alkaline lakes. Chloride and carbonate/bicarbonate, the primary anions and major contributors to osmotic pressure of the lakes (Supplementary Table 1), determine the microbial community composition (Banciu and Muntyan, 2015). Chloride-dominated brines exhibit approximately two times the osmotic pressure of the carbonate-dominated brines with the same Na^+^ molarity (Sorokin et al., 2015; Vavourakis et al., 2018), which explains why the chloride-dominated lakes had a stronger influence on microbial community than the typical (bi)carbonate-rich soda lakes (Zorz et al., 2019).

**FIGURE 2:**
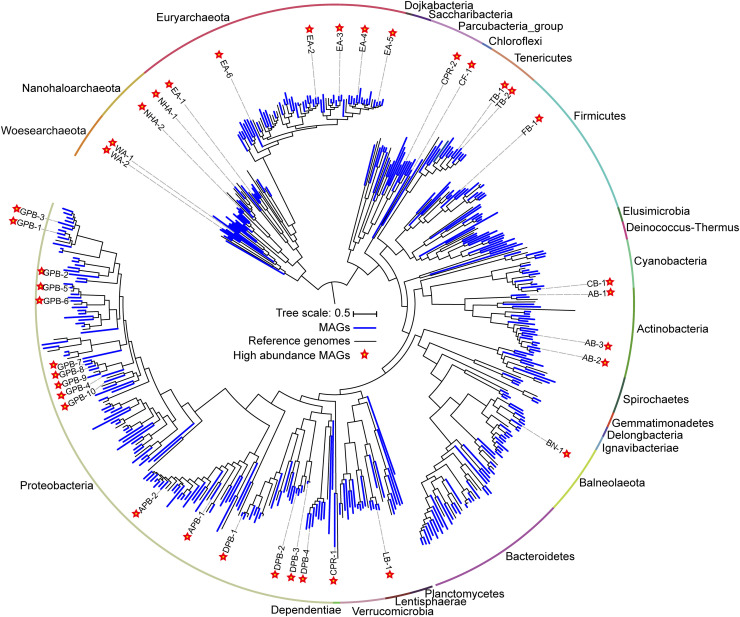
Phylogenomic tree of 385 metagenome-assembled genomes (MAGs) and the evolutionary distribution of abundant MAGs. The MAGs and reference genomes are colored blue and black, respectively. Abundant MAGs are marked with red five-pointed stars. The outer circle is colored by phylum. The complete tree is available with full bootstrap support values in Data Sheet 3.

We further assessed the microbial taxonomic profiles at the phylum level and their relative abundances in each of the samples based on the classification of non-redundant genes by alignment against the NCBI nr database (Figure 1B). Sixteen phyla were present at abundance levels (>0.1%) (Supplementary Table 3). *Proteobacteria* (bacteria) and/or *Euryarchaeota* (archaea) constituted the majority of phyla across the 18 samples. In brine samples, the abundance of *Proteobacteria* ranged from 13.6 to 31.5% in samples HC5W, DK15W, HC17W, HC27W, DK20W, and HC22W with relatively low Cl^–^, whereas this abundance was not greater than 5.5% in water samples containing extremely high Cl^–^ levels (HC26W, DK27W, and DK32W). In contrast, the abundance of *Euryarchaeota* increased from 0.1 to 41.7% along with the increase of Cl^–^ concentrations. *Proteobacteria* and *Euryarchaeota* were also the most abundant phyla in sediment samples. In addition, *Firmicutes*, *Actinobacteria*, *Bacteroidetes*, *Cyanobacteria* and *Ca.* Nanohaloarchaeota were all detected in abundance in soda-saline lakes (Figure 1B). A large variety of microorganisms were observed (Figures 1, 2, Supplementary Table 4), and a number of haloalkaliphilic microorganisms have been reported to be isolated (Grant, 2006; Antony et al., 2013; Sorokin et al., 2015).

### MAGs Revealed Abundant Species

To determine the microbial composition and putative ecological function, contigs are binned into metagenome-assembled genomes (MAGs). We obtained 385 MAGs (completeness > 50% and contamination < 10%), in which 104 near-complete MAGs (completeness > 90% and contamination < 5%) were from 27 archaeal and bacterial phyla (Figure 2, Supplementary Table 4). The naming convention of MAGs is the abbreviation of class (in *Proteobacteria*), phylum (most phyla), or superphylum (CPR) with serial number (Supplementary Table 4). Of the 79 archaeal MAGs, 55 belonged to *Euryarchaeota*, while the remaining belonged to *Ca.* Nanohaloarchaeota (12 MAGs) and *Ca.* Woesearchaeota (12 MAGs). Of the 55 *Euryarchaeota* MAGs, 48 MAGs were from the class *Halobacteria*, and 7 were affiliated with the classes *Methanomicrobia*, *Methanonatronarchaeia*, *Thermoplasmata* and unclassified *Euryarchaeota* (Supplementary Table 4). In bacteria, most MAGs belonged to the phyla *Proteobacteria* (119 MAGs), *Firmicutes* (33 MAGs), *Bacteroidetes* (29 MAGs) and *Actinobacteria* (20 MAGs). Additionally, many other diverse MAGs were obtained, including *Balneolaeota*, *Tenericutes*, *Verrucomicrobia*, *Cyanobacteria*, *Spirochetes*, and CPR (Figure 2, Supplementary Table 4).

**FIGURE 3:**
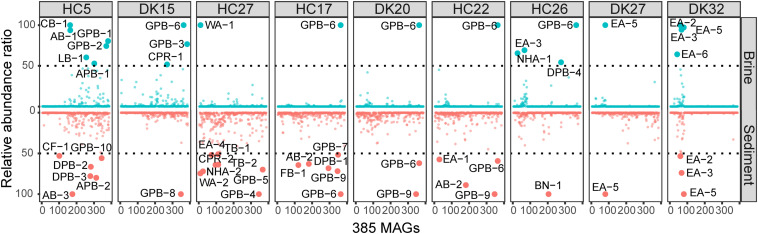
Relative abundances of 385 MAGs in 18 samples. The abundance was estimated by the average depth of all contigs in that MAG (Shown in Supplementary Table 5). The relative abundances of MAGs with the highest sequence read coverage in each sample were scaled to 100, and MAGs with relative abundances of greater than 50 were considered to be abundant. Blue and red indicate the abundances of MAGs in brine and sediment samples, respectively.

**FIGURE 4:**
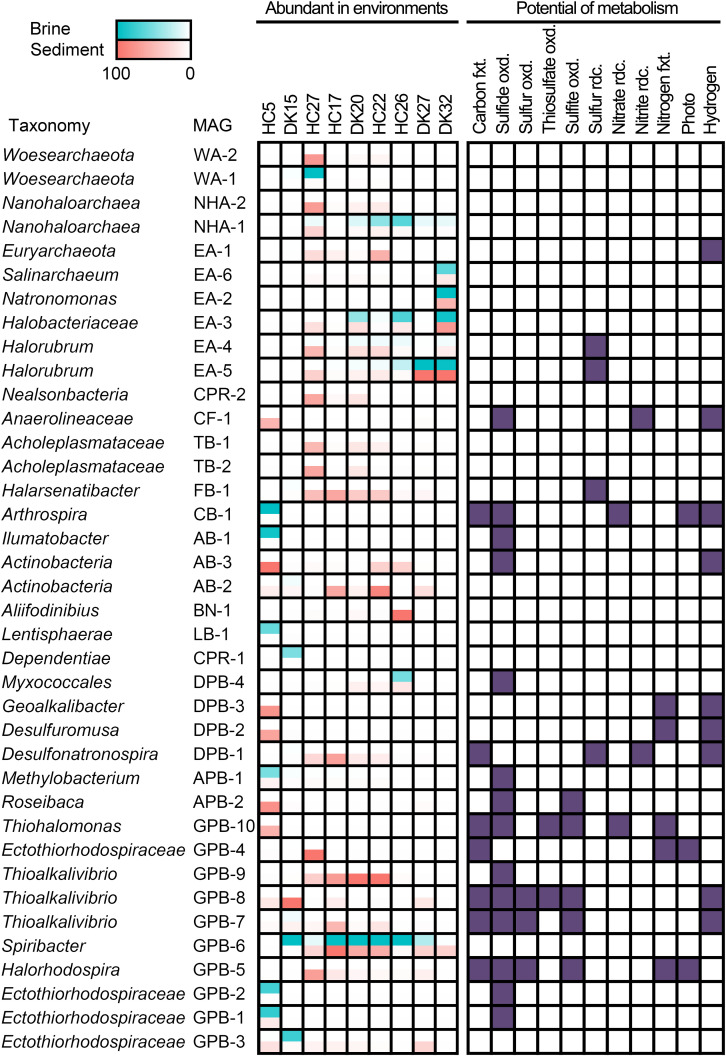
Distribution and metabolic potential of abundant MAGs. Abundant MAGs in brine and sediment samples are colored red and green, respectively; the relative abundance was shown in the legend. MAGs with metabolic potential are colored in purple. fxt., fixation; oxd., oxidation; rdc., reduction.

The relative abundances of the 385 MAGs in the 18 samples are shown in Figure 3. The microbiomes of the 18 brine and sediment samples comprised a small number of abundant species (relative abundance > 50% of MAGs with the highest coverage in the same niche) and a large number of rare MAGs (Figure 3). We observed more abundant bacterial MAGs in brine and sediment samples with relatively low chloride and salinity, especially *Gammaproteobacteria* (*Thioalkalivibrio*, *Spiribacter*, *Thiohalomonas*, and *Halorhodospira*), *Deltaproteobacteria* (*Desulfuromusa* and *Desulfonatronospira*) *Alphaproteobacteria* (*Methylobacterium* and *Roseibaca*), *Actinobacteria* (*Ilumatobacter*), and *Tenericutes*. Cyanobacteria MAG CB-1 was abundant in the brine with the lowest concentration of chloride (Figure 4). Two CPR MAGs were found in abundance, including CPR-1 in DK15W and CPR-2 in HC27S (Figure 4). Some bacterial MAGs were abundant in relatively high chloride samples, such as *Balneolaeota* MAG BN-1 in HC26S, *Deltaproteobacteria* MAG DPB-4 and *Gammaproteobacteria* MAG GPB-6 in HC26W (Figure 4). *Thioalkalivibrio*, *Halorhodospira*, *Desulfonatronospira* and *Roseibaca* were observed in classic soda lakes (Vavourakis et al., 2018). *Thiohalomonas*, *Desulfuromusa*, and *Spiribacter* were usually obtained from neutral saline environments (Vandieken et al., 2006; Lopez-Perez et al., 2013; Mori et al., 2015). Interestingly, GPB-6 (*Spiribacter* sp.) was an abundant microbe in brine and/or sediment samples from five ponds with different salinities (Figure 4), suggesting that GPB-6 exhibited excellent adaptation to saline and alkaline environments. In agreement with this observation, a previous study demonstrated that *Spiribacter salinus* M19-40 is one of the most predominant bacteria in neutral saline lakes (Leon et al., 2014), and the streamlined genome of this bacterium is considered to provide significant advantage in environmental adaptation (Lopez-Perez et al., 2013). This genus was not reported to be abundant in classic soda lakes (Vavourakis et al., 2016, 2018). We also observed archaeal MAGs were abundant in samples with relatively highest salinities, including *Euryarchaeota* (*Salinarchaeum*, *Natronomonas*, *Halorubrum* in *Halobacteriaceae*), *Ca.* Nanohaloarchaeota and *Ca.* Woesearchaeota (Figure 4). In the brine of soda lakes, *Halorubrum*- and *Natrinema*-related sequences were reported to be the most abundant taxa of *Euryarchaeota* (Vavourakis et al., 2016), while *Thermoplasmata* group KTK 4A and *Halobacteria* (*Halohasta* and *Halorubrum*) were detected in the sediment (Vavourakis et al., 2018). Most members of *Salinarchaeum* and *Natronomonas* were isolated from neutral solar salterns (Kamekura et al., 1997; Minegishi et al., 2017). These abundant taxa were the close relatives of microorganisms from both classic soda lakes and neutral saline environments, exhibiting a combination of aforementioned habitats.

A total of 38 abundant MAGs were marked (with five-pointed star) in the phylogenomic tree based on the alignment of universal proteins across *Bacteria* and *Archaea* (Figure 2). Although these abundant MAGs accounted only approximately 10% of the total MAGs, they were from 13 phyla representing half of biodiversity at phylum level (Supplementary Table 7) and accounting for 24.81–69.23% of total coverage of 385 MAGs in 18 samples (Supplementary Table 5). Therefore, these taxa are ideal to understand the microbial functions and adaptation mechanisms in these specific alkaline chloride-carbonate-sulfate niches. To this end, we summarized the metabolic potential of these abundant MAGs in Supplementary Table 6.

### Wide Distribution of Sulfur Oxidation in Autotrophic and Heterotrophic Bacteria

A total of 18 abundant MAGs have the potential to drive the dissimilatory cycling of sulfur element, and sulfide oxidation was present in 14 MAGs. In the autotrophic *Ectothiorhodospiraceae* family, two MAGs (GPB-4 and GPB-5) encoded bacterial photosynthetic reaction center and marker genes (*prk* encoding phosphoribulokinase, *rbcLS* encoding ribulose-bisphosphate carboxylase large and small chain) of the CBB cycle and were both abundant in the sediment of HC27 (Figure 4). Considering the potential for light energy utilization and anoxygenic photosynthetic lifestyle in these species, they may be dominant carbon fixing microbes at the surfaces of sediment. GPB-5 was observed to encode sulfide:quinone oxidoreductase (Sqr), flavocytochrome c sulfide dehydrogenase (FccB), reversed dissimilatory sulfite reductase (rDsrAB), and sulfite dehydrogenase (SoeABC) (Supplementary Table 6), exhibiting the metabolic potential to oxidize multiple sulfur compounds (Figure 4). GPB-5 was predicted to belong to the genus *Halorhodospira* and may be an anoxygenic purple sulfur bacterium. GPB-4 was identified as an unclassified member of the family *Ectothiorhodospiraceae* and did not show the ability to oxidize sulfur compounds or hydrogen (Supplementary Table 6). However, considering that the completeness of the MAG GPB-4 was relatively low (67.44%), partial genes encoding sulfur-oxidizing proteins (SoxBYZ), SoeC, coenzyme F420 hydrogenase subunit beta and hydrogenase expression/formation proteins in GPB-4 may indicate a putative ability of this organism to oxidize sulfur compound or hydrogen (Supplementary Table 6). Notably, both GPB-4 and GPB-5 possessed the nitrogenase gene *nifHDK* (Supplementary Table 6), indicating that they may be involved in nitrogen fixation in pond HC27. Two *Thioalkalivibrio* MAGs (GPB-7 and GPB-8) and one *Thiohalomonas* MAG (GPB-10) were abundant in sediment samples from ponds HC17, DK15 and HC5, respectively (Figure 4). These three MAGs had marker genes for the CBB cycle and the oxidization of multiple sulfur compounds (Supplementary Table 6), which is consistent with the metabolic characteristics of *Thioalkalivibrio* and *Thiohalomonas*. Apart from carbon fixation and sulfur cycling, GPB-7 and GPB-8 contained hydrogenase genes and may utilize hydrogen as energy source and reducing power, while GPB-10 harbored *nifHDK* genes (Supplementary Table 6) and exhibited the potential to fix nitrogen (Figure 4). In brief, our results suggest that carbon fixing and sulfur oxidation may be coupled in these anoxygenic photosynthetic and chemolithotrophic *Ectothiorhodospiraceae* species.

In addition, eight cyanobacterial MAGs were obtained, while only one MAG (CB-1) belonging to the cyanobacterial genus *Arthrospira* was detected as an abundant species in the brine of HC5 (Supplementary Table 4), which had the lowest salinity and chloride concentration (Figure 4). Cyanobacteria are typically the primary contributors to carbon fixation. CB-1 encoded Prk, RbcLS and the complete photosystems II and I (Supplementary Table 6), indicating that it has the potential to photosynthetically fix carbon via the CBB cycle (Figure 4). This MAG also encoded hydrogenase, nitrate reductase, and Sqr, exhibiting versatile metabolic capabilities. Four other cyanobacterial MAGs had the potential to perform photosynthesis, of which three encoded Sqr and two encoded nitrogenase (Supplementary Table 6). It was interesting to note that under alkaline conditions, most photosynthetic *Cyanobacteria* MAGs had the potential to oxidize sulfide (Supplementary Table 6) and may regulate photosynthesis and carbon fixing (Klatt et al., 2015). In addition, some cyanobacteria have been reported to be anoxygenic photosynthetic bacteria and capable of oxidizing sulfide by Sqr (Cohen et al., 1975; Sybesma et al., 1986; Grim and Dick, 2016; Hamilton et al., 2018). The electrons obtained from sulfide oxidation are further transferred to the reducing equivalent NADPH via quinone and electron transport chain components and are finally used for CO_2_ fixation (52).

Interestingly, we observed that many abundant bacteria from heterotrophic taxa also harbored Sqr, including AB-1 and AB-3 from *Actinobacteria*, CF-1 from *Chloroflexi*, APB-1 and APB-2 from *Alphaproteobacteria*, DPB-4 from *Deltaproteobacteria*, and GPB-1, GPB-2, and GPB-9 from *Gammaproteobacteria* (Figures 2, 4), indicating their potential in oxidation-mediated detoxification of sulfide with various salinities in the saline-alkaline environments. In addition, four *Halobacteria* MAGs EA-2, EA-4, EA-5, and EA-6 contain the thiosulfate dehydrogenase [quinone] large subunit (DoxD, KEGG Orthology: K16936). The UniProt list reports a wrong annotation (Vavourakis et al., 2019), but the lack of the small subunit (DoxA) suggested that the ability for thiosulfate oxidation might be absent. Sulfide and thiosulfate could provide accessory energy under nutrient-limited conditions to heterotrophs and increase the growth rate and flux of assimilatory carbon via anaplerotic reaction of oxaloacetate. This lifestyle is considered to be facultative lithoheterotrophy (Sorokin, 2003). Alkaline lakes, including soda-saline lakes (Figure 4) and typical soda lakes (Sorokin and Kuenen, 2005; Tourova et al., 2013), provide excess soluble phosphate and a low toxic form of sulfide (HS^–^), creating an advantage environment for the growth of diverse sulfur-oxidizing microbes (Sorokin et al., 2015; Vavourakis et al., 2018). The *sqr* gene was found in an abundance of heterotrophic taxa, such as *Actinobacteria*, *Chloroflexi*, *Alphaproteobacteria*, *Deltaproteobacteria*, and *Gammaproteobacteria*, suggesting that these microbes play a role in the detoxification of sulfide. Interestingly, a large number of strictly organotrophic microbes inhabiting soda lakes or marine environments are capable of oxidizing thiosulfate to tetrathionate or sulfate (Sorokin, 2003; Sorokin et al., 2005). An increased growth rate of *Limnobacter thiooxidans*, a thiosulfate-oxidizing heterotrophic bacterium isolated from freshwater lake sediment, was observed by adding thiosulfate, suggesting that *L. thiooxidans* obtains an energy advantage via the oxidation of thiosulfate (Spring et al., 2001). Heterotrophic sulfur-oxidizing microbes (HSOB) provided thiosulfate restored the ATP synthesis in the starved cells (Sorokin, 2003) and increased the level of dark anaplerotic carbon dioxide assimilation (Tuttle and Jannasch, 1977; Perez and Matin, 1982). Since ATP synthesis is a rate-limiting step for the anaplerotic CO_2_ assimilation, the energy advantage obtained from sulfide oxidation by Sqr could be a reasonable explanation for the high coverage of Sqr-containing heterotrophs.

### Potential Reduction of Sulfur Compounds in Abundant MAGs

Given that numerous autotrophic and heterotrophic abundant MAGs exhibited the potential to oxidize reductive sulfur compounds (RSCs), it would be interesting to investigate whether there were considerable sulfur-reducing microbes to complete the sulfur cycling in the chloride-carbonate-sulfate lakes. Notably, we found the presence of anaerobic respiration processes in abundant MAGs, including sulfur respiration (Figure 4). *Halorubrum* spp. EA-4, EA-5, *Halarsenatibacter* sp. FB-1, *Desulfonatronospira* sp. DPB-1 harbored the *psrA/phsA* gene (Supplementary Table 6), allowing for potential polysulfide reduction/thiosulfate reduction. The *Desulfonatronospira* MAG DPB-1 was an abundant species in the sediment of HC17 (Figure 4) and encoded thiosulfate reductase/polysulfide reductase chain A (*psrA/phsA*) and F420-non-reducing hydrogenase subunits (*mvhADG*) (Supplementary Table 6), indicating the lifestyle of chemolithotrophic sulfate-reducing bacterium. It is noteworthy that DPB-1 also had the marker genes of anaerobic carbon-monoxide dehydrogenase catalytic subunit (*cooS*), acetyl-CoA synthase (*acsB*), and acetyl-CoA decarbonylase/synthase subunits (*cdhDE*) in the WL pathway (Supplementary Table 6), indicating that it may fix inorganic carbon via the WL pathway.

In addition to elemental sulfur reduction, we also observed the potential of sulfate/sulfite and tetrathionate reduction in many MAGs. Seven MAGs from *Desulfobacterales* and *Desulfovibrionales* harbored the *dsrAB* gene for sulfate/sulfite respiration, and some of them had the potential for carbon fixing (Supplementary Table 6). The type strains from same taxonomies were reported facultative autotrophs using hydrogen and/or formate as substrates (Lien et al., 1998; Pikuta et al., 2003; Sorokin et al., 2008a, b; Sorokin and Chernyh, 2017). *Halomonas* sp. GPB-59, *Marinobacter* sp. GPB-11, and *Aquisalimonas* sp. GPB-38 harbored *ttrABC* genes for tetrathionate reductase, while another five MAGs from *Actinobacter* and one *Desulfuromusa* MAG had *ttrAB* (Supplementary Table 6) with the potential of tetrathionate respiration. Although the abundance was not high, all the above taxa could drive sulfur reduction and make sulfur cycling complete.

Interestingly, many elemental sulfur-reducing microbes, such as *Halorubrum* spp. EA-4, EA-5, *Halarsenatibacter* sp. FB-1, and *Desulfonatronospira* sp. DPB-1 (Figure 4), were abundant under extremely hypersaline conditions, given that the much lower concentrations of dissolved oxygen (Sherwood et al., 1991) and the notably reduced oxygen diffusion coefficients (Jamnongwong et al., 2010) boosted the anaerobic respiration of these microbes. In addition, diverse taxa were involved in sulfate and tetrathionate respiration (Supplementary Table 6). Using acetate as a substrate, the polysulfide/elemental sulfur reduction under alkaline conditions (pH 10) is much more exergonic (**ΔG =** -91.9 kJ/mol) than neutral pH conditions (**ΔG^0^**′** =** -6.6 kJ/mol) (Reaction 1 in Table 1), suggesting that this reaction is energetically more favorable for dissimilatory elemental sulfur reduction at a higher pH value (Sorokin et al., 2010). However, for those microbes utilizing heterotrophic and chemolithotrophic sulfate respirations, approximately no differences in free energy changes are noted between neutral and alkaline conditions. Given that polysulfide/elemental sulfur-reducing microbes are able to obtain more free energy for ATP synthesis under alkaline conditions (Table 1), the difference in ATP yields between the two pH conditions could explain why polysulfide/elemental sulfur-reducing microbes could gain a growth advantage in alkaline environments.

**TABLE 1:** Summary of Gibbs free energy change in sulfur reactions^#^.

Rct. No.	Chemical reaction	ΔG^0^′ (kJ)	ΔG^0^ at pH 10 (kJ)	References
1	Acetate^–^ + 4S^0^ + 4H_2_O → 4HS^–^ + 2HCO_3_^–^ + 5H^+^	−6.6	−91.9	Oren, 2011
2	Acetate^–^ + 4S_4_O_6_^2–^ + 4H_2_O → 2HCO_3_^–^ + 9H^+^ + 8S_2_O_3_^2–^	−233.4	−386.94	Thauer et al., 1977
3	Acetate^–^ + SO_4_^2–^ → 2HCO_3_^–^ + HS^–^	−47.3	−47.3	Thauer et al., 1977
4	4H_2_ + SO_4_^2–^ + H^+^ → HS^–^ + 4H_2_O	−151.9	−134.84	Thauer et al., 1977

*^#^The ΔG values were calculated using acetate or hydrogen as samples and presented as the Gibbs free energy change per reaction. Eight electrons were transferred in every reaction.*

### Symbionts Exist Abundant in Hypersaline Environments

Abundant MAGs, including 2 *Ca.* Nanohaloarchaeota MAGs and 2 *Ca.* Woesearchaeota MAGs in the DPANN superphylum as well as 1 *Ca.* Dependentiae MAG and 1 *Ca.* Nealsonbacteria MAG in the CPR superphylum, were also observed in these saline-alkaline lakes (Figures 2, 4). The metabolic potential of these microbes is typically limited due to the smaller genome size (Supplementary Table 4). All six MAGs did not show the potential for dissimilatory sulfur or nitrogen metabolism (Figure 4). Interestingly, most DPANN and CPR taxa are auxotrophic with respect to the biosynthesis of amino acids, purine and pyrimidine bases of nucleotides, and isoprenoids or fatty acids of the cell membrane (Castelle et al., 2018). The two *Acholeplasmataceae* MAGs TB-1 and TB-2 were observed to be abundant in the sediment of HC27 (Figure 4). *Acholeplasmataceae* is a class of cell-wall-free microbes (fried egg-like colony) that live together with plants or insects (Freundt et al., 1984).

**FIGURE 5:**
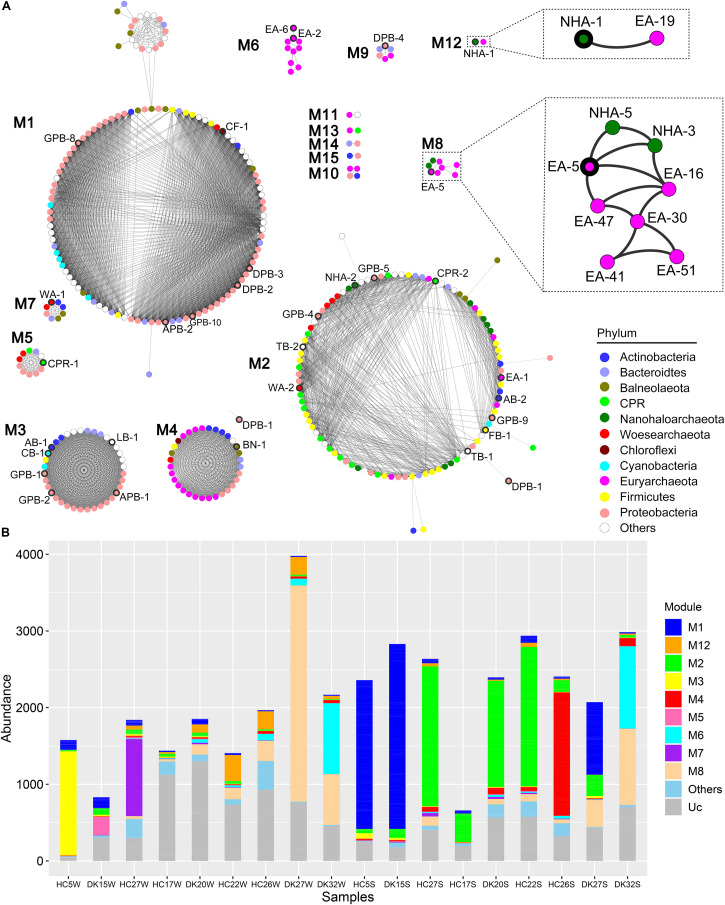
Co-occurrence network based on all 385 MAGs. **(A)** The coverage of 385 MAGs in 18 samples was used to construct a robust co-occurrence network (Pearson correlation coefficient > 0.9 and *p*-value < 0.01). The nodes in the network are colored by phylum, and the nodes representing abundant MAGs are labeled and bold in the outer ring. Two modules involved are zoomed in to show the robust co-occurrence of *Candidatus* Nanohaloarchaeota and *Halobacteria*. **(B)** The abundance of MAGs from the modules was summarized in different sample group. Other, sum of M9, M10, M11, M13, M14, and M15; Uc, unclassified in any module.

Members of the DPANN and CPR superphyla have symbiotic lifestyles with other microbes (Castelle et al., 2018; Hamm et al., 2019). We constructed a co-occurrence network based on the coverage of 385 MAGs in the 18 samples to predict the putative associated symbiont (Figure 5). There were fifteen separate modules named M1 to M15 in the network (Figure 5A). M1, M2, and M4 contained most MAGs and existed almost in sediment samples, while M3, M5, M7, and M12 were observed in brine samples. MAGs in M6 and M8 were mainly found in hypersaline brine or sediment samples (Figure 5B). Among fifteen modules, only one abundant *Ca.* Nanohaloarchaeota MAG (NHA-1) was strongly co-present with the *Halobacteriaceae* MAG EA-19 in M12, while NHA-5 and NHA-3 correlated with *Natronomonas* sp. EA-16 and the abundant *Halorubrum* sp. EA-5 in M8, as well as with each other (Figure 5A). The above results suggested that the putative symbiont of *Ca.* Nanohaloarchaeota is likely a member of the taxon *Halobacteria*.

*Ca.* Nanohaloarchaeota and other DPANN members are difficult to culture in the laboratory (Castelle and Banfield, 2018; Liu et al., 2018; Hamm et al., 2019) given that their small sizes of genomes limit their anabolic abilities (Narasingarao et al., 2012; Castelle et al., 2018). Generally, these microbes still need to uptake nutrients from their symbionts for the biosynthesis of nucleic acids, proteins and lipids. *Ca.* Nanohaloarchaeum antarcticus was isolated together with *Halorubrum lacusprofundi* but away from other species (Hamm et al., 2019). The growth of *Ca.* Nanohaloarchaeota is probably dependent on a specific *Halobacteria* host (Hamm et al., 2019) (Figure 5), increasing the difficulty of culturing *Ca.* Nanohaloarchaeota. Although few *Ca.* Nanohaloarchaeota species have been cocultured with their hosts, they are abundant in multiple hypersaline environments, including neutral salterns (Ghai et al., 2011; Narasingarao et al., 2012), soda lakes (Vavourakis et al., 2016), and soda-saline lakes (Figures 2, 4). The “salt in” strategy *Ca.* Nanohaloarchaeota employs to resist osmotic force (Vavourakis et al., 2016) provides an effective solution of saving energy in hypersaline environments (Banciu and Muntyan, 2015; Gunde-Cimerman et al., 2018). This energy-saving mechanism may contribute to the observed high abundance of *Ca.* Nanohaloarchaeota in hypersaline environments.

### Metabolic Advantage of *Ca.* Nanohaloarchaeota in Hypersaline Environments

**FIGURE 6:**
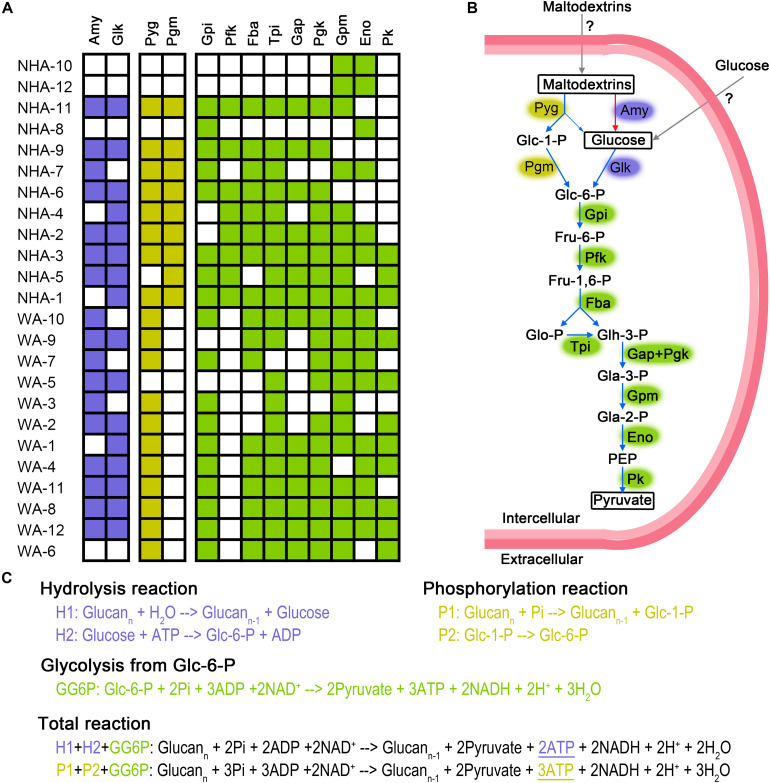
Putative fermentation pathways in *Candidatus* Nanohaloarchaeota and *Candidatus* Woesearchaeota MAGs. The presence of enzymes is exhibited in detailed **(A)**. We draw the metabolic pathway of carbohydrate fermentation in *Ca.* Nanohaloarchaeota **(B)**. Blue and red arrows represented the presence or absence, respectively, of that pathway in abundant MAG NHA-1. Gray arrow indicates that the specific transporters were not identified. Biochemical reaction and ATP production of glycolysis via phosphorylation and hydrolysis were compared **(C)**. The metabolic potential of the phosphorylation, hydrolysis and glycolysis pathways are colored purple, yellow and green, respectively. Amy, alpha-Amylase; Glk, Glucokinase; Pyg, Glycogen phosphorylase (or named 1,4-alpha-Glucan phosphorylase); Pgm, Phosphoglucomutase; Gpi, Glucose-6-phosphate isomerase; Pfk, 6-Phosphofructokinase; Fba, Fructose-bisphosphate aldolase; Tpi, Triosephosphate isomerase; Gap, Glyceraldehyde 3-phosphate dehydrogenase; Pgk, Phosphoglycerate kinase; Gpm, Phosphoglycerate mutase; Eno, Enolase; Pk, Pyruvate kinase; Glc-1-P, Glucose 1-phosphate; Glc-6-P, Glucose 6-phosphate; Fru-6-P, Fructose 6-phosphate; Fru-1,6-P, Fructose 1,6-biphosphate; Glo-P, Glycerone phosphate; Glh-3-P, Glyceraldehyde 3-phosphate; Gla-3-P, Glycerate 3-phosphate; Gla-2-P, Glycerate 2-phosphate; PEP, Phosphoenolpyruvate; ATP, Adenosine 5’-triphosphate; ADP, Adenosine 5’-diphosphate; NAD^+^, Nicotinamide adenine dinucleotide; NADH, Reduced nicotinamide adenine dinucleotide; Pi, phosphate.

The presence of symbiont must create mutually beneficial relationships; otherwise, the symbiont would be replaced by a separate existence. We constructed the energy generation pathway in DPANN superphylum to deeply understand the symbiotic lifestyle. DPANN and CPR cannot regenerate ATP via oxidative and photosynthetic phosphorylation due to the general absence of electron transport complexes and photosynthetic reaction center complexes (Supplementary Table 6). Some taxa (especially NHA-1 and NHA-3) are believed to gain energy by substrate level phosphorylation via carbohydrate fermentation for the complete glycolysis pathway from hexose to pyruvate (Figures 6A,B). *Ca.* Woesearchaeota in saline-alkaline lakes may ferment carbohydrates via a modified pathway or generate energy through another pathway considering the absence of 6-phosphofructose kinase (pfkAB or pfkC) (Figure 6A and Supplementary Table 6). Pyruvate:ferredoxin oxidoreductase, alcohol dehydrogenase and lactate dehydrogenase were not detected in *Ca.* Nanohaloarchaeota MAGs (Supplementary Table 6), indicating that pyruvate may be produced as final product for the symbiotic microbes (potentially *Halobacteria*). Consistently, no *Halobacteria* MAGs contained the polysaccharide phosphorylation pathway (Supplementary Table 6). Pyruvate is a key nutrient in hypersaline environments (Oren, 2015), indicating that the *Ca.* Nanohaloarchaeota and *Ca.* Woesearchaeota taxa may function as primary degraders of polysaccharides (Figure 6A) at least in the symbiotic system.

Most DPANN MAGs contained alpha-amylase and glucose kinase; thus, the 1,4-alpha-glucans (like starch) may be generally used as their carbon and energy sources (Figure 6A). Interestingly, eight of twelve *Ca.* Nanohaloarchaeota MAGs had genes encoding glycogen phosphorylase (or named 1,4-alpha-glucan phosphorylase) and phosphomannomutase/phosphoglucomutase enzymes (Pgm), which catalyze the phosphorylation of polysaccharides and transfer glucose-1-phosphate to glucose-6-phosphate (Figure 6B). In contrast, more *Ca.* Woesearchaeota MAGs encoded glycogen phosphorylase, but none of them harbored the *pgm* gene (Figure 6A). More interestingly, the abundant MAG NHA-1 contained 1,4-alpha-glucans phosphorylase and a complete glycolysis pathway, but not alpha-amylase (Figure 6A). Given the increased ATP produced compared with hydrolysis (Figure 6C), we infer that 1,4-alpha-glucans phosphorylation may play a significant role in maintaining the symbiotic lifestyle between *Ca.* Nanohaloarchaeota sp. NHA-1 and its *Halobacteria* host (discuss below).

**FIGURE 7:**
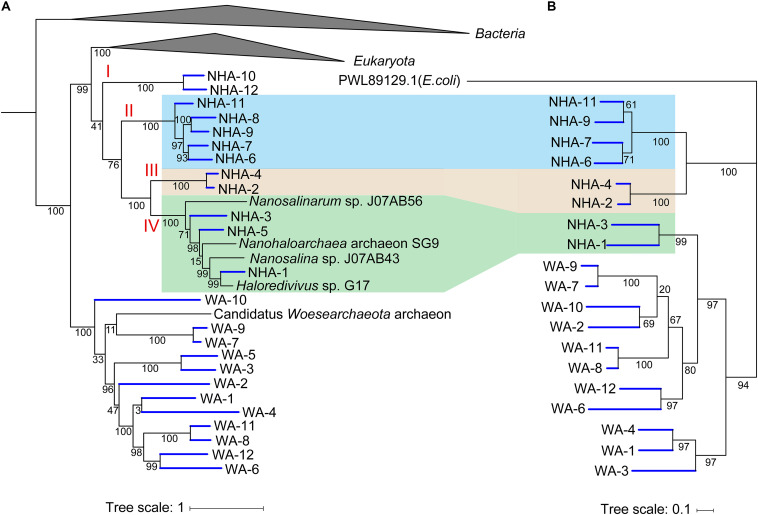
Phylogenetic analysis of amino acid sequences of glycogen phosphorylases obtained from *Candidatus* Nanohaloarchaeota and *Candidatus* Woesearchaeota MAGs. **(A)** The tree shown to the left is the phylogenomic tree of 385 MAGs with the reference genome, and the *Bacteria* and *Euryarchaeota* branches are collapsed. **(B)** Nineteen predicted glycogen phosphorylases in *Ca.* Nanohaloarchaeota and *Ca.* Woesearchaeota with the alpha-glucan phosphorylase from *Escherichia* coli (PWL89129.1) were retrieved, aligned and then used to infer the correct tree using the Maximum Likelihood method based on the JTT matrix-based model with MEGA. The tree with the highest log likelihood (-16393.43) is shown with bootstrap support.

To estimate the importance of 1,4-alpha-glucans phosphorylation, we compared the similarity of functional genes among DPANN. In the phylogenomic tree, four separate clades in *Ca.* Nanohaloarchaeota were observed (Figure 7A). The glycogen phosphorylase coding gene pyg was generally detected in clades II, III and IV but was not present in NHA-10 and NHA-12 from clade I (Figure 6A). Among the separate clades, Pyg from different MAGs with a high similarity were located together (Figure 7B), especially in NHA-1 and NHA-3. Interestingly, the amino acid sequences of the glycogen phosphorylase from clades II and III were located at closed branches, while that of clade IV was homologous with the enzyme from *Ca.* Woesearchaeota (Figure 7B). The evolutionary tree of Pyg indicated that phosphorylation was conserved in most taxa of *Ca.* Nanohaloarchaeota.

*Ca.* Nanohaloarchaeota utilizes polysaccharide and generates ATP by substrate level phosphorylation during glycolysis (Vavourakis et al., 2016; Castelle et al., 2018). Alpha-amylase could be responsible for the hydrolysis of polysaccharide (Vavourakis et al., 2016; Castelle et al., 2018; Liu et al., 2018). More recently, *Ca.* Nanahalobium has been proven to be capable of hydrolyzing alpha-glucans (La Cono et al., 2019). Amylase genes were widely distributed in *Ca.* Nanohaloarchaeota and *Ca.* Woesearchaeota (Figure 6A), but they are absent in NHA-1, the most abundant *Ca.* Nanohaloarchaeota strain in hypersaline environments. The 1,4-alpha-glucan phosphorylation pathway, which is involved in maltose/maltodextrin/glycogen metabolism in both archaea and bacteria (Boos and Shuman, 1998; Xavier et al., 1999; Seibold et al., 2009), could be employed by the NHA-1 strain as an alternative strategy for polysaccharide degradation (Figures 6A,B). However, both 4-alpha-glucanotransferase catalyzing the conversion of maltose to maltodextrin and glycogen synthase responsible for the synthesis of glycogen were not available in NHA-1 (Supplementary Table 6). Therefore, we hypothesize that extracellular maltodextrin is a putative substrate. One less ATP molecule is used when one glucoside molecule is degraded via phosphorylation pathway rather than the hydrolysis pathway, so one more ATP molecule will be made by using maltodextrin substrate (Figure 6C). This relatively more efficient ATP generation system present in *Ca.* Nanohaloarchaeota MAG may provide the symbionts with a growth advantage in the competition with the free-living *Halobacteria*, which typically utilize starch via the alpha-amylase-based hydrolysis pathway (Perez-Pomares et al., 2003).

## Conclusion

Chloride-carbonate-sulfate lakes (also known as soda-saline lake) is a double-extreme environment with high pH and high salinity, and diverse metabolic processes function well in such an environment. This study has revealed the microbial composition and their metabolic potential in the brines and sediments of the chloride-carbonate-sulfate lakes. The microbiomes from different habitats were composed of several abundant and numerous rare taxa. These abundant taxa represented most branches of the phylogenomic tree. The oxidation and reduction of sulfur and polysaccharide phosphorylation existed in certain abundant taxa, which may increase their adaptation to extreme alkaline and saline environments with unique advantages in terms of energy production and thermodynamics. Briefly, RSCs could be utilized as a putative accessory energy source for heterotrophs under nutrient limited conditions. Elemental sulfur respiration more easily occurs under high pH may due to thermodynamic advantages, which favors this type of sulfur reduction microbes in high abundance. More energy was produced by the phosphorylation pathway of 1,4-alpha-glucans compared with hydrolysis. The above results provide novel insights into the relationship between diverse lifestyles and adaptive characterizations of the prokaryotes thriving in such double-extreme environments.

## Data Availability Statement

The datasets generated for this study can be found in the NCBI (projectID PRJNA549802), gcMeta (projectID NMDC10010899), and figshare (https://figshare.com/s/9c3cb76f0c9646a30e94).

## Author Contributions

HX designed and supervised the study. DZ, SZ, QX, JC, JZ, FC, and HX collected water and sediment samples. QX and JC measured physicochemical characteristics. YZ extracted DNA from environmental samples. DZ and SZ performed bioinformatic and statistical analyses under the partial supervision of HY and SH. DZ and SZ prepared the figures and wrote the manuscript under the guidance of HX. ML, YZ, SL, and SH participated in discussions and revisions. All authors read and approved the final manuscript.

## Conflict of Interest

The authors declare that the research was conducted in the absence of any commercial or financial relationships that could be construed as a potential conflict of interest.

## Footnotes

1 https://github.com/cjfields/readfq

2 https://sourceforge.net/projects/bbmap/

3 http://ecogenomics.github.io/BamM

4 https://www.ncbi.nlm.nih.gov/

5 https://www.r-project.org/

6 http://vegan.r-forge.r-project.org/

7 https://CRAN.R-project.org/package=ggpubr

8 https://CRAN.R-project.org/package=pheatmap

9 https://github.com/vqv/ggbiplot

10 https://CRAN.R-project.org/package=psych

11 https://figshare.com/s/9c3cb76f0c9646a30e94
