# Supplementary figures and images for: Abundant Taxa and Favorable Pathways in the Microbiome of Soda-Saline Lakes in Inner Mongolia
Source: Front Microbiol. 2020 Jul 24;11:1740. doi: 10.3389/fmicb.2020.01740 (PMC7393216; doi:10.3389/fmicb.2020.01740)

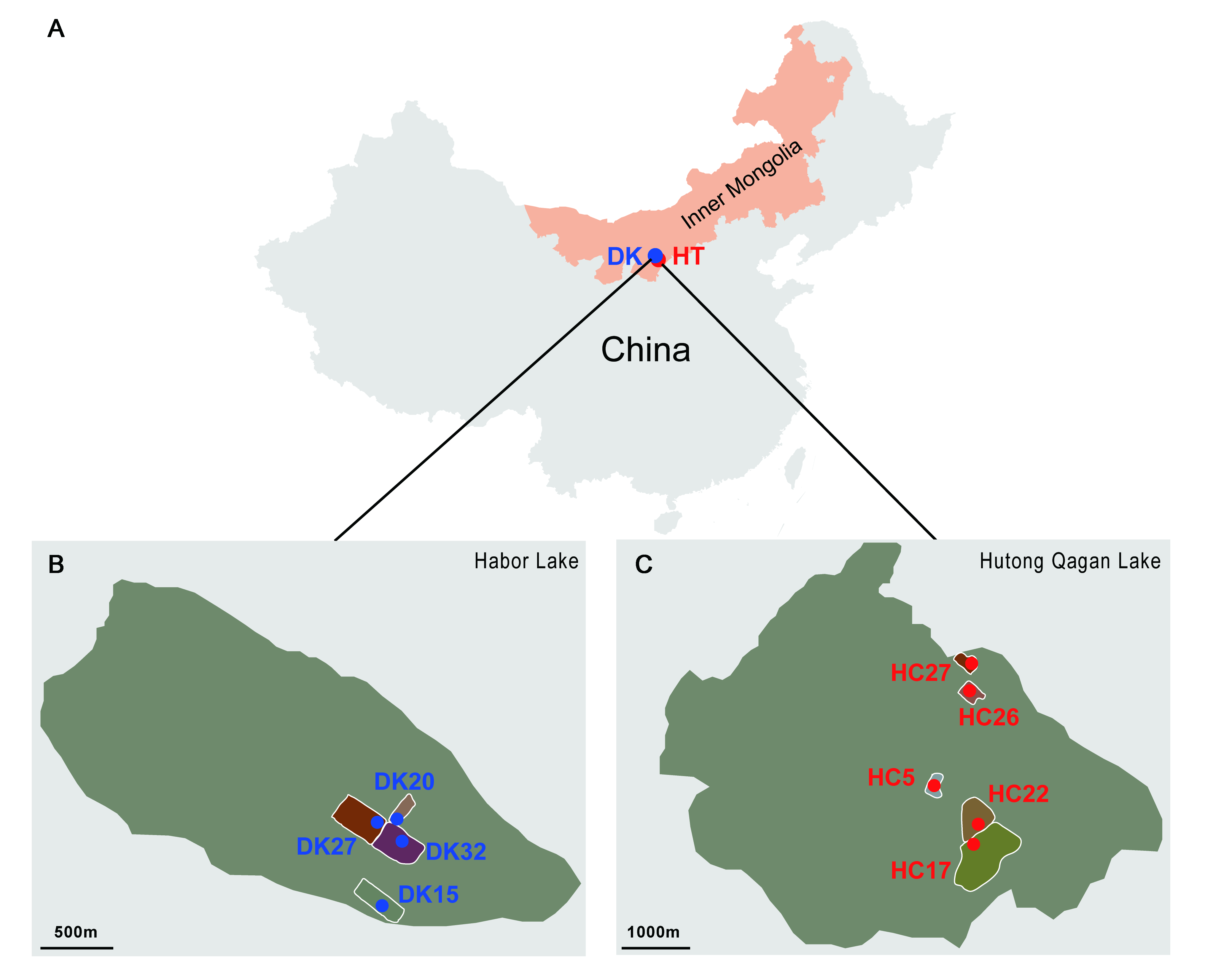

Supplement: Supplementary file 1 [file Image_1.TIF]

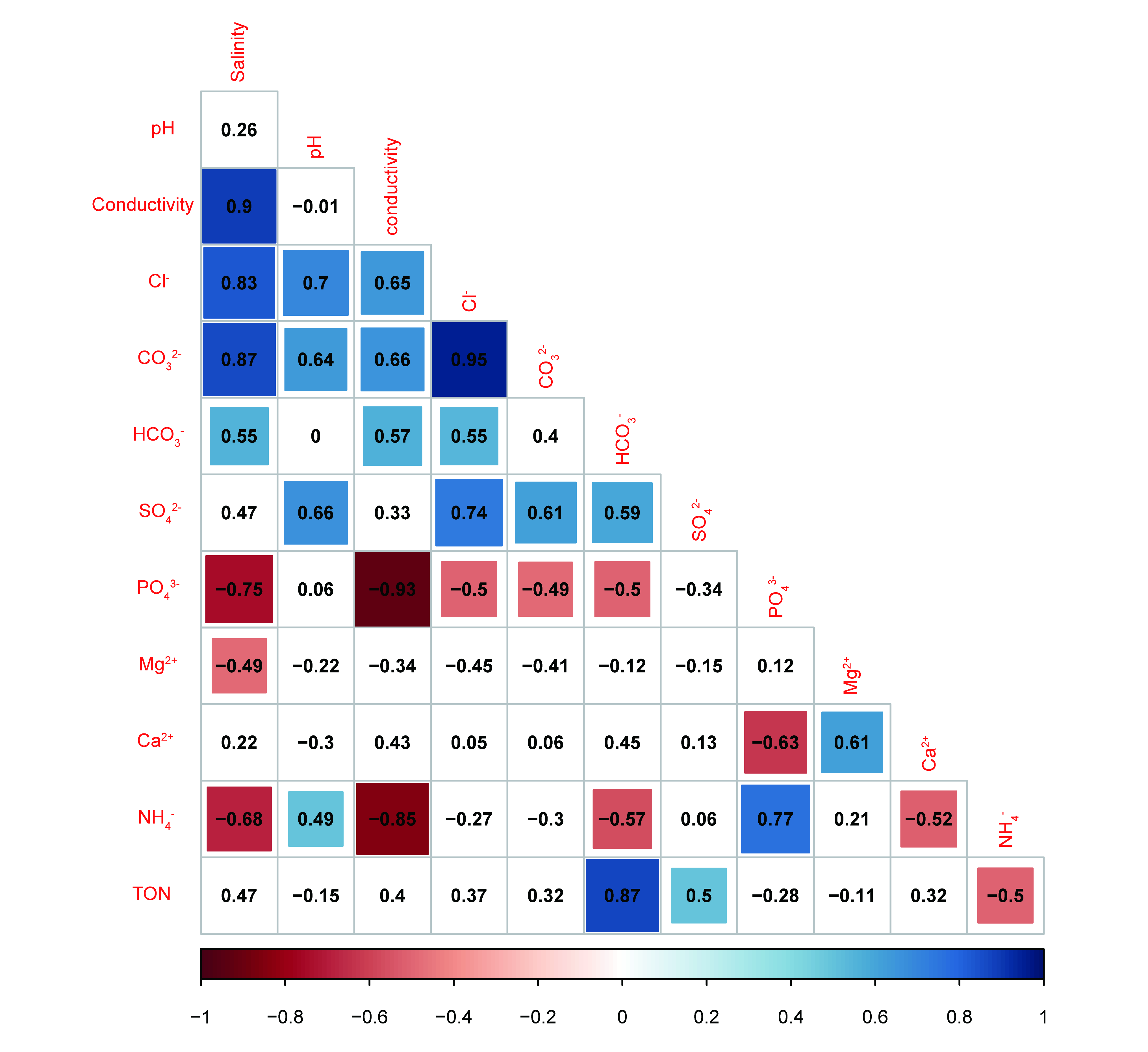

Supplement: Supplementary file 2 [file Image_2.TIF]

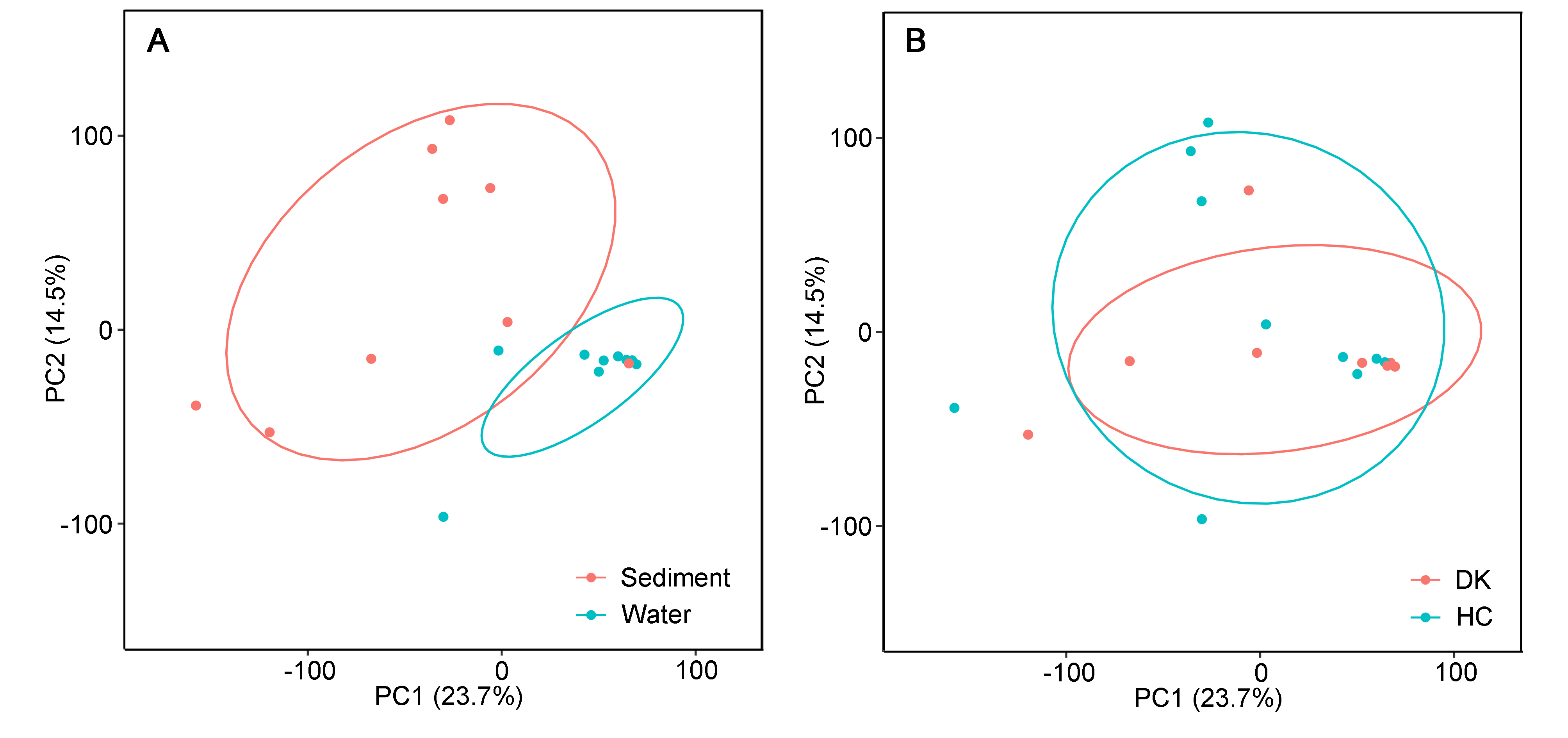

Supplement: Supplementary file 3 [file Image_3.TIF]

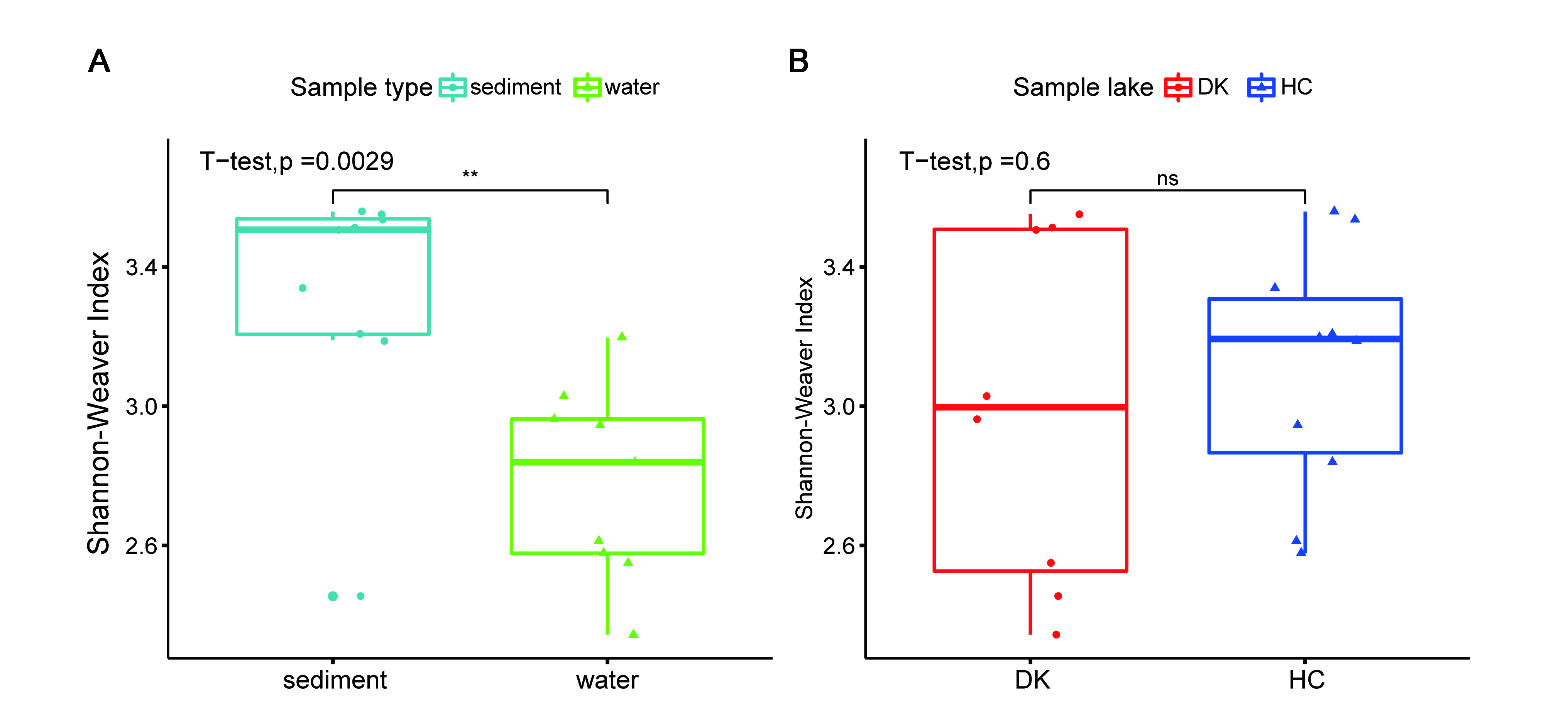

Supplement: Supplementary file 4 [file Image_4.TIF]
