# Supplementary material for: Abundant Taxa and Favorable Pathways in the Microbiome of Soda-Saline Lakes in Inner Mongolia
Source: Front Microbiol. 2020 Jul 24;11:1740. doi: 10.3389/fmicb.2020.01740 (PMC7393216; doi:10.3389/fmicb.2020.01740)
Supplement: Supplementary file 6 [file Data_Sheet_1.DOCX]

Supplementary Material

# Notes for Data Sheets

**Data Sheets 1** Introduction to supplementary materials (this files).

**Data Sheets 2** Abundance of each metagenomic operational taxonomic unit in eighteen samples.

**Data Sheets 3** Complete 385 MAGs phylogenomic tree in newick format.

**Data Sheets 4** Functional annotation of genes from 385 MAGs.

# Legends for Supplementary Images

**Supplementary Image 1** Locations of Hutong Qagan Lake (HT) and Habor Lake (DK) in the Inner Mongolia Autonomous Region, China (A), and the locations of sampling sites in DK (B) and HT (C). DK and HC are 35 kilometers apart.

**Supplementary Image 2** Correlation between 11 environmental factors. The correlation matrix was calculated using Pearson’s correlation. The numbers in the squares represent the correlation coefficients, which are equal to the proportion of the fill color in each square. Only significant correlations (p-value < 0.05) were filled with a corresponding color.

**Supplementary Image 3** Influence of water/sediment (A) and different lakes (B) on microbial community structure was analyzed by principal component analysis. The ellipses were 68% confidence interval.

**Supplementary Image 4** Comparison of α-diversity between 18 samples. The Shannon-Weaver Index was calculated and compared across different sample types (A) and sample lakes (B). Significances were calculated by T-test.

# Legends for Supplementary Tables

**Supplementary Table 1** Environmental factors of ponds of Hutong Qagan Lake and Habor Lake

**Supplementary Table 2** General informations of metagenomes of eighteen samples

**Supplementary Table 3** The microbial composition and relative abundance at phylum level (>0.1%)

**Supplementary Table 4** General description and taxonomy of 385 high-quality MAGs

**Supplementary Table 5** The coverages of 385 MAGs in eighteen samples

**Supplementary Table 6** Metabolic potentials of carbon, sulfur and nitrogen in 385 MAGs

**Supplementary Table 7** The number of MAGs in phylum level

**Supplementary Table 8** Determination of direction of sulfur metabolism by DsrAB
